# Ex vivo mesoscopic diffusion MRI correlates with seizure frequency in patients with uncontrolled mesial temporal lobe epilepsy

**DOI:** 10.1002/hbm.25139

**Published:** 2020-07-21

**Authors:** Justin Ke, Lesley M. Foley, T. Kevin Hitchens, R. Mark Richardson, Michel Modo

**Affiliations:** ^1^ Department of Radiology University of Pittsburgh Pittsburgh Pennsylvania USA; ^2^ Department of Bioengineering University of Pittsburgh Pittsburgh Pennsylvania USA; ^3^ Animal Imaging Center University of Pittsburgh Pittsburgh Pennsylvania USA; ^4^ Department of Neurobiology University of Pittsburgh Pittsburgh Pennsylvania USA; ^5^ Centre for the Neural Basis of Cognition University of Pittsburgh Pittsburgh Pennsylvania USA; ^6^ Department of Neurological Surgery Massachusetts General Hospital Boston Massachusetts USA; ^7^ Harvard Medical School Boston Massachusetts USA; ^8^ McGowan Institute for Regenerative Medicine Pittsburgh Pennsylvania USA

**Keywords:** biomarker, connectivity, diffusion mri, epilepsy, hippocampus, mesoscale, surgical resection, tractography

## Abstract

The role of hippocampal connectivity in mesial temporal lobe epilepsy (mTLE) remains poorly understood. The use of ex vivo hippocampal samples excised from patients with mTLE affords mesoscale diffusion magnetic resonance imaging (MRI) to identify individual cell layers, such as the pyramidal (PCL) and granule cell layers (GCL), which are thought to be impacted by seizure activity. Diffusion tensor imaging (DTI) of control (*n* = 3) and mTLE (*n* = 7) hippocampi on an 11.7 T MRI scanner allowed us to reveal intra‐hippocampal connectivity and evaluate how epilepsy affected mean (MD), axial (AD), and radial diffusivity (RD), as well as fractional anisotropy (FA). Regional measurements indicated a volume loss in the PCL of the cornu ammonis (CA) 1 subfield in mTLE patients compared to controls, which provided anatomical context. Diffusion measurements, as well as streamline density, were generally higher in mTLE patients compared to controls, potentially reflecting differences due to tissue fixation. mTLE measurements were more variable than controls. This variability was associated with disease severity, as indicated by a strong correlation (*r* = 0.87) between FA in the stratum radiatum and the frequency of seizures in patients. MD and RD of the PCL in subfields CA3 and CA4 also correlated strongly with disease severity. No correlation of MR measures with disease duration was evident. These results reveal the potential of mesoscale diffusion MRI to examine layer‐specific diffusion changes and connectivity to determine how these relate to clinical measures. Improving the visualization of intra‐hippocampal connectivity will advance the development of novel hypotheses about seizure networks.

## 1 INTRODUCTION

Mesial temporal lobe epilepsy (mTLE) affecting the hippocampus is the predominant form of focal seizure disorders (Bertram, 2009). Although there are effective treatments that control seizure activity in most patients, some experience refractory epilepsy that is currently not controlled using pharmacological means (Kwan & Brodie, 2000). In these cases, surgical removal of the ictal locus might be recommended to reduce or completely alleviate seizure activity (Yasargil, Krayenbuhl, Roth, Hsu, & Yasargil, 2010). Although semiology and electroencephalography (EEG) remain criticial to define the ictal locus, magnetic resonance imaging (MRI) is increasingly playing a key role in localizing the area for resection, as well as monitoring adaptive post‐operative changes in surrounding tissues (Engel & International League Against, 2001; Thom, Mathern, Cross, & Bertram, 2010). From a radiological perspective, mTLE often presents with hippocampal atrophy and a hyperintense T_2_ signal (Cendes, 2013). MRI volumes in sub‐fields are correlated with neuronal density, whereas increases in T_2_‐weighted signal intensities are associated with gliosis (Goubran et al., 2016). These biomarkers are generally considered unspecific downstream pathological events, rather than putative causative events, such as changes in cellularity in hippocampal layers (Reddy, Younus, Sridhar, & Reddy, 2019; Wiest & Beisteiner, 2019). Although changes in connectivity due to Mossy fiber sprouting have been hypothesized to form a reverberant excitatory network, evidence of this aberrant connectivity in humans remains sparse (Buckmaster, 2014; Modo, Hitchens, Liu, & Richardson, 2016; Scharfman, 2019).

Diffusion MRI can assess microstructural properties of tissue, such as cellularity, myelination, and axonal damage based on mean, radial and axial diffusivity (Seehaus et al., 2015). Diffusion‐based imaging of fiber tracts is also increasingly being applied to understand macroscopic networks underlying seizure activity (Alizadeh et al., 2019; Bao et al., 2018), including hippocampal subfield specific tractography (Rutland et al., 2018). In some cases, an enhanced connectivity per hippocampal voxel was evident (Dinkelacker et al., 2015). The cornu ammonis (CA) 1, dentate gyrus and subiculum have been suggested as functional network hubs that subserve seizure activity (Shah et al., 2018). A high resolution study of excised hippocampi at 7 T afforded the identification of the pyramidal cell layer (PCL) and indicated that increased mean diffusivity (MD) was characteristic of hippocampal sclerosis (Coras, Milesi, et al., 2014). A major challenge that remains is to resolve intra‐hippocampal structures and their networks to evaluate how these contribute to seizure activity. There is generally a good correspondence between hippocampal cell layers observed on high resolution MRI and histology (Adler et al., 2018; Coras, Milesi, et al., 2014; Ly et al., 2020; Modo et al., 2016; Schoene‐Bake et al., 2014; Wieshmann et al., 1999). High spatial resolution imaging at the mesoscale (0.1–1 mm) is required to resolve hippocampal layers and sub‐fields (Modo et al., 2016; Rondinoni, Magnun, Vallota da Silva, Heinsen, & Amaro Jr., in press).

To achieve a mesoscale resolution and dissect fiber tracts connecting different layers of the hippocampus, ex vivo imaging is currently required due to the long scanning time required to acquire high‐resolution 3‐dimensional images with sufficient diffusion‐encoding directions (Ly et al., 2020). We previously determined that, for instance, the granule cell layer (GCL) of the dentate gyrus (DG) is only 0.2 mm thick, hence requiring at least a resolultion of 0.1 mm to achieve coverage by 2 imaging voxels for tractography (Ly et al., 2020; Modo et al., 2016). Due to the laminar organization of the hippocampus along its axis, prior studies have achieved excellent images with very high in‐plane resolution (Coras, Milesi, et al., 2014; Shepherd, Ozarslan, Yachnis, King, & Blackband, 2007). However, the disproportionaly low‐resolution slice thickness precludes a robust and valid tracing of fiber tracts connecting different hippocampal layers. An isotropic voxel dimension of 0.1 mm is required to probe intra‐hippocampal connectivity (Ly et al., 2020). A major advantage of ex vivo samples is that extended scanning times can be implemented to achieve sufficient signal‐to‐noise (SNR) at a mesoscale resolution (Beaujoin et al., 2018; Coras, Milesi, et al., 2014; Dell'Acqua, Bodi, Slater, Catani, & Modo, 2013; Ly et al., 2020; Modo et al., 2016). As there is a high correspondence between in vivo and ex vivo measurement of the hippocampal formation (Wisse et al., 2017), ex vivo investigations are advantageous to discover novel imaging targets, as these avoid motion artifacts, afford longer scanning times, permit repeat scanning of the same sample and allow histological comparisons for validation of imaging results (Rondinoni et al., in press).

The objective of this study was to determine cell layer specific diffusion changes in resected hippocampi from patients with intractable mTLE at a 0.1 mm isotropic resolution, while probing network changes using tractography. Scalar diffusion indices, consisting of mean (MD), radial (RD) and axial diffusivity (AD), as well as fractional anisotropy (FA), were measured for the PCL in CA1‐4, GCL of the DG, stratum moleculare (SM), stratum radiatum (SR) and stratum oriens (SO) in hippocampi from patients with mTLE and post‐mortem controls. Tractography revealed intra‐hippocampal connections between different layers, affording a quantitation of connectivity and demonstration of “aberrant” connectivity between GCL and SM. Our investigation here demonstrates that ex vivo mesoscopic diffusion MRI of excised hippocampi from epileptic patients can provide unique insights into seizure networks. This approach can potentially bridge the gap between histopathological analyses and diagnostic radiology.

## 2 MATERIALS AND EQUIPMENT

### 2.1 Patient selection and sample preparation

This study was approved by the Institutional Review Board at the University of Pittsburgh. Subjects were patients assessed by a multidisciplinary epilepsy board, who were determined to have pharmaco‐resistant mesial temporal lobe epilepsy (mTLE), as a result of concordant neuroimaging, neuropsychiatric and electrophysiological data, and who underwent anterior temporal lobectomy following the recommendation of the clinical team. Specimen excision was achieved via *en bloc* hippocampectomy (Kucukyuruk, Richardson, Wen, Fernandez‐Miranda, & Rhoton Jr., 2012; Yasargil et al., 2010). A total of 13 patient samples were enrolled, of which seven samples from patients were included for analysis here (Table 1). The posterior body of the hippocampus was made available for the imaging research study, with the remainder being used for the standard clinical neuropathological evaluation of patients. A total of six samples were excluded out of the 13 patients that were enrolled. Samples of <800 mm^3^ were excluded from the study (*n* = 3), as these typically only afforded a segmentation of the GCL of the DG, but did not afford a robust delineation of other regions of interest (ROIs). Additional exclusion criteria consisted of seizure frequency > 10/month (*n* = 2) or pediatric onset of disease (*n* = 1), as these were not considered representative of the experimental group. The average age of patients was 44 years (range 23–68) with a mean disease length of 18.4 years (range 2–50) and a disease burden of 4.8 seizures/month (range 1–8). Excised hippocampi were transferred into 4% formaldehyde and post‐fixed for 48 hr before being transferred to phosphate buffered saline (PBS) for storage at 4°C. To provide anatomical context, control hippocampi (*n* = 3) were obtained post‐mortem from patients with an average age of 63 (range 60–65) that did not have brain damage at the time of death. Time to fixation after death was 19–20 hr. Hippocampi were immersed into 10% buffered formalin (CH_2_O equivalent to 4% formaldehyde) for 6 weeks at 4°C prior to transfer to PBS.

**TABLE 1:** Patient characteristics

#	Age	Sex	Side	Duration (years)	Frequency (per month)	MRI findings	Pathology summary	Surgical outcome at last followup visit	
								Engel class	Months post‐op
1	38	F	R	20	4	Hippocampal T2 hyperintensity and volume loss	Almost complete loss of pyramidal cells in CA1, CA3/4. Minimal loss of granule cell layer in dentate gyrus. Diffuse gliosis and microglia activation	IIIA	34
2	36	F	R	15	8	Subtle hippocampal T2 hyperintensity	Selective CA3 neuron loss. Hippocampal sclerosis. Diffuse gliosis with variable microglia activation	IA	60
3	68	F	R	17	4	Hippocampal T2 hyperintensity and volume loss	Moderate neuron loss in CA4. Decrease in granule cells in dentate gyrus. Gliosis in CA4	IIC	44
4	45	M	L	8	8	Hippocampal T2 hyperintensity and volume loss	Progressive hypocellularity of layers. Small segments lacking neurons. Hippocampal sclerosis. Extensive gliosis	IIB	8
5	23	M	L	2	1	Subtle hippocampal T2 hyperintensity	No clear neuronal distinction of pyramidal (i.e., CA‐1‐4) and granule cell layer (i.e., dentate gyrus). No evidence of hippocampal sclerosis	IB	36
6	38	F	R	12	5	Non‐lesional	Neuron loss in CA4 and granule cell layer of dentate gyrus. Hippocampal sclerosis. Gliosis in CA4	1A	30
7	60	F	R	50	4	Non‐lesional	Hippocampal sclerosis	1A	22

*Note*: The Engel epilepsy surgery outcome scale has the following classes—IA: Completely seizure‐free since surgery, IB: Non disabling simple partial seizures only since surgery, IC: Some disabling seizures after surgery, but free of disabling seizures for at least 2 years, ID: Generalized convulsions with antiepileptic drug withdrawal only, IIA: Initially free of disabling seizures but has rare seizures now; IIB: Rare disabling seizures since surgery, IIC: More than rare disabling seiuzres after surgery, but rare seizures for at least 2 years, IID: Nocturnal seizures only, IIIA: Worthwhile seizure reduction, IIIB: Prolonged seiuzre‐free intervals amounting to greater than half the follow‐up period, but not less than 2 years, Class IV: No worthwhile improvement.

### 2.2 MRI scanning

For MR scanning (<1 month post‐excision), samples were immersed in proton‐free FluorInert (Sigma), while avoiding the generation of air bubbles in a syringe that afforded immobilization of the specimen to reduce motion artifacts. Samples were placed in an 11.7 T/89 mm Bruker Avance AV3 HD microimaging scanner with a Micro 2.5 gradient insert (capable of up to 150 G/cm). A 20 mm diameter quadrature birdcage RF coil was used to accommodate the diameter of the 10 ml syringe. Scanning parameters were defined in Paravision 6.0.1 (Bruker Biospin, Billerica, MA), which was used to acquire images. High resolution anatomical reference 3D T_2_‐weighted Spin Echo image series were acquired with 16 equally spaced echoes (repetition time, TR = 4,000 ms; echo time, TE = 10 ms; number of averages, NEX = 1; field of view, FOV = 25.6 × 12.8 × 12.8 mm; matrix = 256 × 128 × 128; 100 μm isotropic resolution; 8 hr 52 min scanning time). T_2_ maps were computed for signal measurements. Using the same geometry as the T_2_‐weighted images, DTI images were acquired with a 3D Pulsed Gradient Spin Echo (PGSE) sequence (TR = 1,100 ms, TE = 25 ms, diffusion duration *δ* = 4 ms, diffusion spacing Δ = 15 ms, diffusion time *t*_D_ = 13.6 ms, 12 non‐colinear diffusion directions, *b*‐value = 4,000, 100 μm isotropic resolution; 63 hr scanning time) (Ly et al., 2020). Sample temperature control was achieved with a Bruker SmartCooler BCU‐1 40/50 air chiller and a probe heater with a thermocouple feed‐back loop to maintain the sample temperature to within ±0.1°C. Sample temperature was maintained at 8 ± 0.1°C to minimize sample degradation and provide constant temperature for diffusion measurements. The data that support the findings of this study are available from the corresponding author upon reasonable request.

### 2.3 Diffusion image preprocessing

Diffusion MR images were processed using DSI Studio (available at http://www.dsistudio.labsolver.org) (Yeh, Verstynen, Wang, Fernandez‐Miranda, & Tseng, 2013). The sample was masked using a signal threshold to remove background prior to processing. No upsampling or motion correction was used. Reconstruction of the diffusion tensor images (DTI) was achieved by performing an Eigenvector analysis on the calculated tensor (Jiang, van Zijl, Kim, Pearlson, & Mori, 2006). Scalar indices of diffusion, notably fractional anisotropy (FA), mean diffusivity (MD), radial diffusivity (RD), and axial diffusivity (AD) were calculated (Basser, Mattiello, & LeBihan, 1994) to determine how epilepsy affected hippocampal tissue microstructure.

### 2.4 Segmentation of hippocampal lamina and subfields

Mean diffusivity (MD) images yielded the most robust contrast between hippocampal cell layers and were thus utilized to manually segment intra‐hippocampal structures (Ly et al., 2020; Modo et al., 2016). The GCL of the DG produced a hyperintense signal that was easily identified in all slices and used to define this region of interest (ROI). The PCL also was defined by a higher signal intensity than the adjacent stratum oriens (SO) and radiatum (SR). The stratum moleculare (SM) was defined in relation to the SR, which had a lower signal intensity, and the GCL, which has a markedly higher signal intensity. The stratum lacunare (SL) could not be distinguished from the SM. Anatomical annotations were based on Duvernoy et al. (Duvernoy, Cattin, & Risold, 2013) and histological studies (Ding & Van Hoesen, 2015; Zeineh et al., 2017), as well as the MR literature on defining hippocampal subfields (Adler et al., 2014; Dalton, Zeidman, Barry, Williams, & Maguire, 2017; Iglesias et al., 2015; Pipitone et al., 2014; Wisse et al., 2012; Yushkevich et al., 2015), recently reviewed in Giuliano et al. (Giuliano et al., 2017). Hippocampal subfields CA1, CA2, CA3, and CA4 were manually defined for the PCL, as these were the focus for connectivity analyses. The CA1/subiculum border was defined as a perpendicular straight line drawn across from the start of the hippocampal sulcus. The CA1/CA2 border was established at the half‐way point of the external limb of the GCL, whereas the CA2/CA3 transition was considered a straight line extending from the end of the external limb of the GCL to the alveus enlarging into the fimbria. The CA3 region was delineated as the region spanning the start of the fimbria emerging from the alveus to the end of the fimbria. A straight line across from the end of the fimbria specified the border between CA3/CA4. The hyperintense region of the PCL extending into the hilus was defined as CA4. The PCL of CA1‐CA3 was a clear hyperintense band, whereas CA4 presented with a mushroom‐like morphology that emerged as a hyperintense band from CA3, but fanned out morphologically mimicking the GCL.

### 2.5 Tractography

Fiber tract reconstructions of the whole specimen were performed within DSI Studio by utilizing a local multi‐direction deterministic Euler fiber‐tracking algorithm (Yeh et al., 2013). Tractography was achieved using the following parameters: 10 seeds/voxel with random sub‐voxel positioning and trilinear interpolation in all orientations, fractional anisotropy thresholded to 0.02, angular threshold of 60°, a 0.05 mm step size (half the voxel length), minimum length = 0.2 mm (twice the voxel length), maximum length = 50 mm, smoothing = 0.2, Otsu threshold = 0.6, and thread count of 12. The total number of streamlines were recorded. To account for volume differences, streamline density (i.e., streamlines divided by volume) was calculated.

### 2.6 Statistics

All data were graphed and analyzed in Prism v8.02 (GraphPad Software, San Diego, CA). No plotting of standard error or statistical comparison between control and epileptic data was performed, considering the inherent differences in tissue fixation that question the face validity of a sample comparison. Control data is hence presented here to provide anatomical context only. To determine the impact of clinical variables, such as age, disease length and severity, Pearson correlations were calculated to determine how these variables affect MR measurements on epilpetic samples. Effect sizes were determined to be neglible (*r* < 0.3), weak (*r* = 0.3–0.5) medium (*r* = 0.5–0.7) or strong (*r* > 0.7) (Mukaka, 2012). Statistical signficance was set to *p* < .05. To account for multiple comparsions in the correlation analyses between different ROIs, a False Discovery Rate (FDR) was computed in Prism and set at *q* < 0.05.

## 3 RESULTS

### 3.1 Magnetic resonance (MR)‐histology of the human hippocampus

**FIGURE 1:**
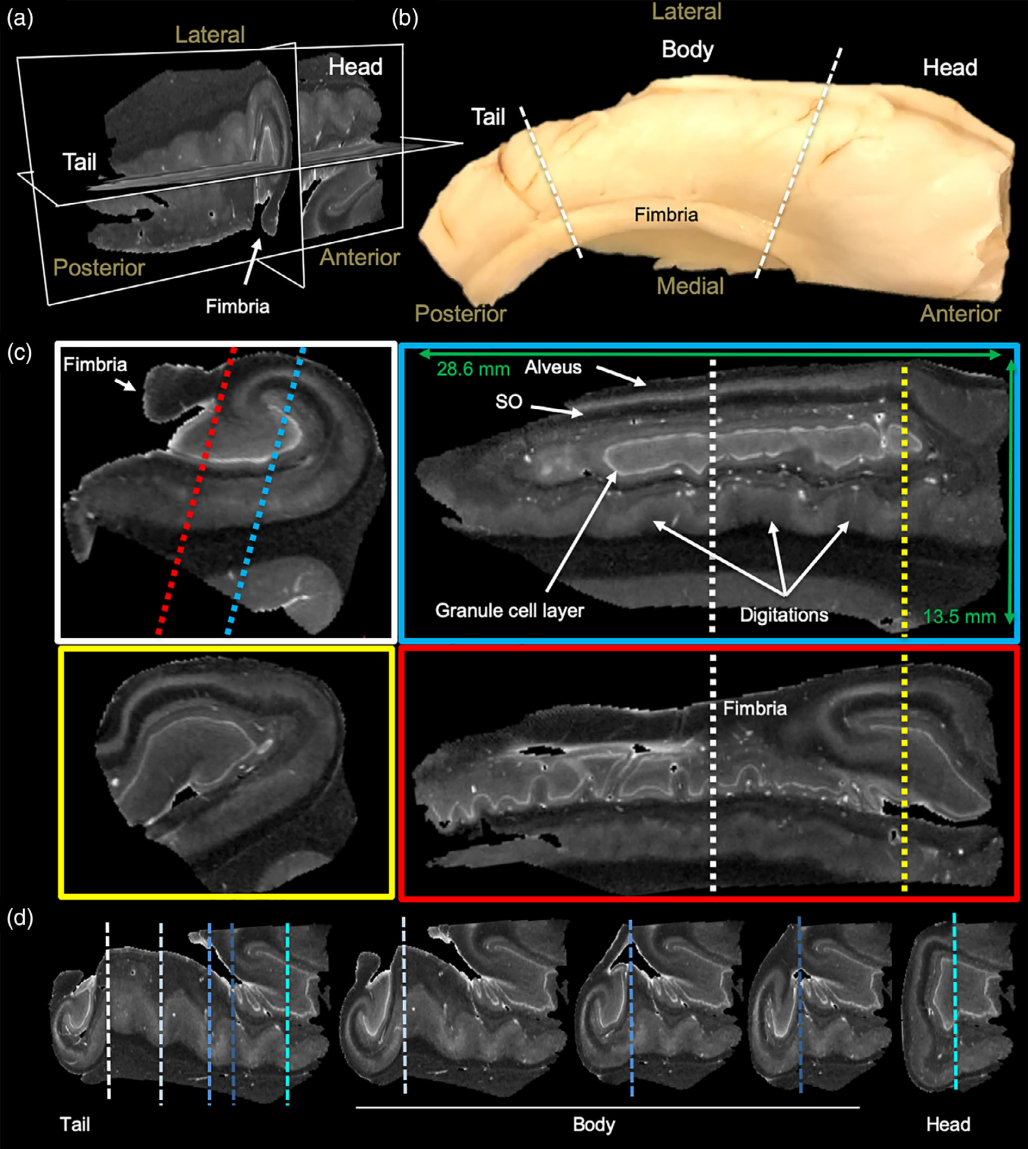
High resolution MR imaging of a whole healthy human hippocampus. (a) Visualization of the three axes of a 100 μm isotropic mean diffusion (MD) image. (b) Photograph of excised hippocampal sample and delineation of the tail, body and head regions. (c) Coronal and saggital sections of the hippocampus reveal signal differences between cell layers with the granule cell layer being more hyperintense than other regions. The stratum oriens (SO) and alveus are easily discernible in the saggital plane. (d) Anterior–posterior comparison of coronal slices

Ex vivo MR imaging of the human hippocampus affords the acquisition of a 3‐dimensional internal anatomy view (Figure 1a) that cannot be gained from an intact specimen (Figure 1b). The fimbria is easily identified to define the medial and lateral side of the hippocampus. The non‐diseased adult whole hippocampus is approximately 28.6 mm long with the head aspect being 13.5 mm thick (Figure 1c). Within this structure, MD maps reveal a very detailed anatomy at 0.1 mm isotropic resolution. Notably, the hyperintense nature of the GCL of the DG is the most prominent feature that helps to orient the anatomical composition. The coronal plane provides a classical view of hippocampal anatomy in which the GCL is identified as a C or a reversed C. The fimbria is also a key anatomical feature to define the medial part of the hippocampus.

Saggital sections along the anterior–posterior (tail‐head) direction reveal a very different anatomical view, with the GCL showing indentations at different parts and being a closed loop. An overlay of MD and FA maps provides further contrast in the transverse (Figure S1a) and saggital planes (Figure S1b) to refine the segmentation of individual neighboring cell layers. MD is highest in the GCL, whereas FA is very low in the GCL and the adjacent layers consisting of the polymorphic layer and SM (Figure S1c). FA is higher in SR and SO, but low again in the PCL. As the PCL has also a higher MD than the adjacent layers, this clearly defines the borders between cell layers. Although the basic arrangement of cell layers is fairly consistent along the tail and body, shape differences in the GCL and other layers are evident along the digitations (Figure 1d). The head region presents a more complex anatomical organization than the body and tail region. The GCL is no longer a C or reversed C, but is a more elongated structure in the saggital plane and in a coronal view presents a closed loop. Cell layers are perpendicular to the organization in the body and tail, as evident at multiple levels in the saggital (Figure S2a) and transverse plane (Figure S2b). The fimbria emerges from the CA3 area in the head region and expands over the body and tail region.

**FIGURE 2:**
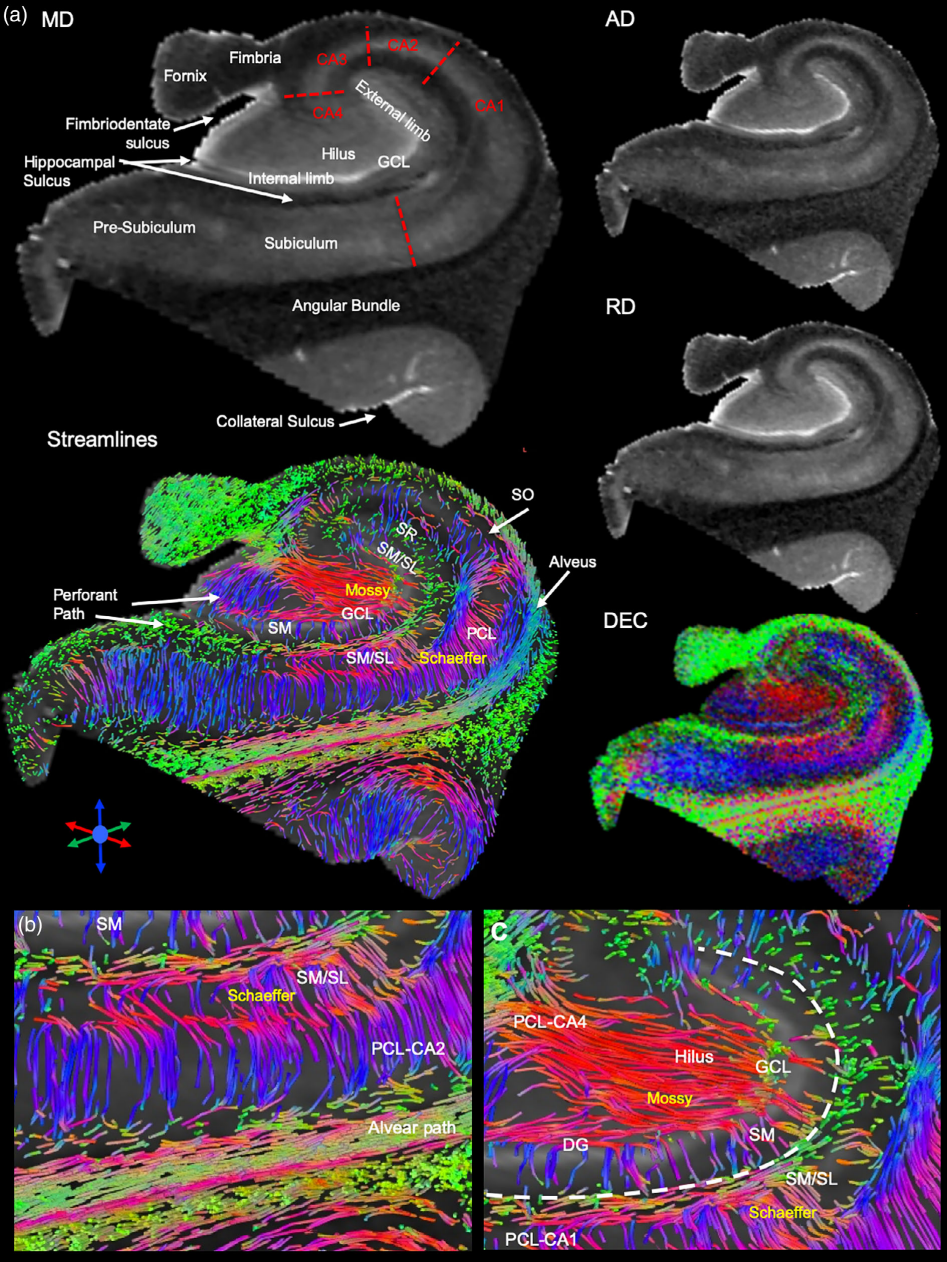
Diffusion MR imaging and regional connectivity. (a) Scalar indices consisting of mean diffusion (MD), axial diffusion (AD) and radial diffusion (RD) define cell layers, such as the granule cell layer (GCL) of the dentate gyrus (DG), as well as the pyramidal cell layer (PCL) that can be sub‐divided in cornu ammonis (CA) 1–4 regions. Tractography of the sample and diffusion encoded color (DEC) images of the fractional anisotropy (FA) further reveal fiber connections between different cell layers. (b) Tractography affords a further dimension of anatomical information that is not discernable from scalar index images that affords the assessment of intra‐hippocampal connectivity. (c) A detailed view of connections, including Mossy fibers and Schaeffer collalerals, afford a system's analysis between different hippocampal layers and regions

We here focused on the anatomical organization of the posterior body and tail region of the hippocampus, as these regions were available for analysis from epileptic patients. In these regions, the GCL of the DG helps define hippocampal architecture, especially on MD images, with subtle differences evident in RD and AD images (Figure 2a). All major hippocampal landmarks are readily identified and afford definition of the PCL for sub‐fields CA1‐4, as well as the fimbria, hippocampal sulcus, subiculum, and angular bundle. Diffusion encoded color (DEC) images provide very distinct and complimentary information that aid in the definition of regions, such as the hilus, SO and alveus. Generation of streamlines from the FA image further refines the anatomy evident on structural images. The perforant and alvear path, the connections between GCL to CA3 (i.e., Mossy fibers), as well as projections from the PCL (i.e., Schaeffer collaterals) are visible. It is noteworthy that in some cases Schaeffer collaterals trace through the PCL and SM/SL, whereas in others, the projections terminate along the SM/SL border (Figure 2b). These Schaeffer collateral projections typically do not merge with the streamlines that define the alvear path, but run perpendicular to these. In the dentate gyrus, Mossy fiber connectivity between the GCL and CA4 is readily identified throughout the hilus region (Figure 2c). Streamlines also emanate from the GCL to project through the SM layer.

### 3.2 Diffusion MR of epileptic and control hippocampi

**FIGURE 3:**
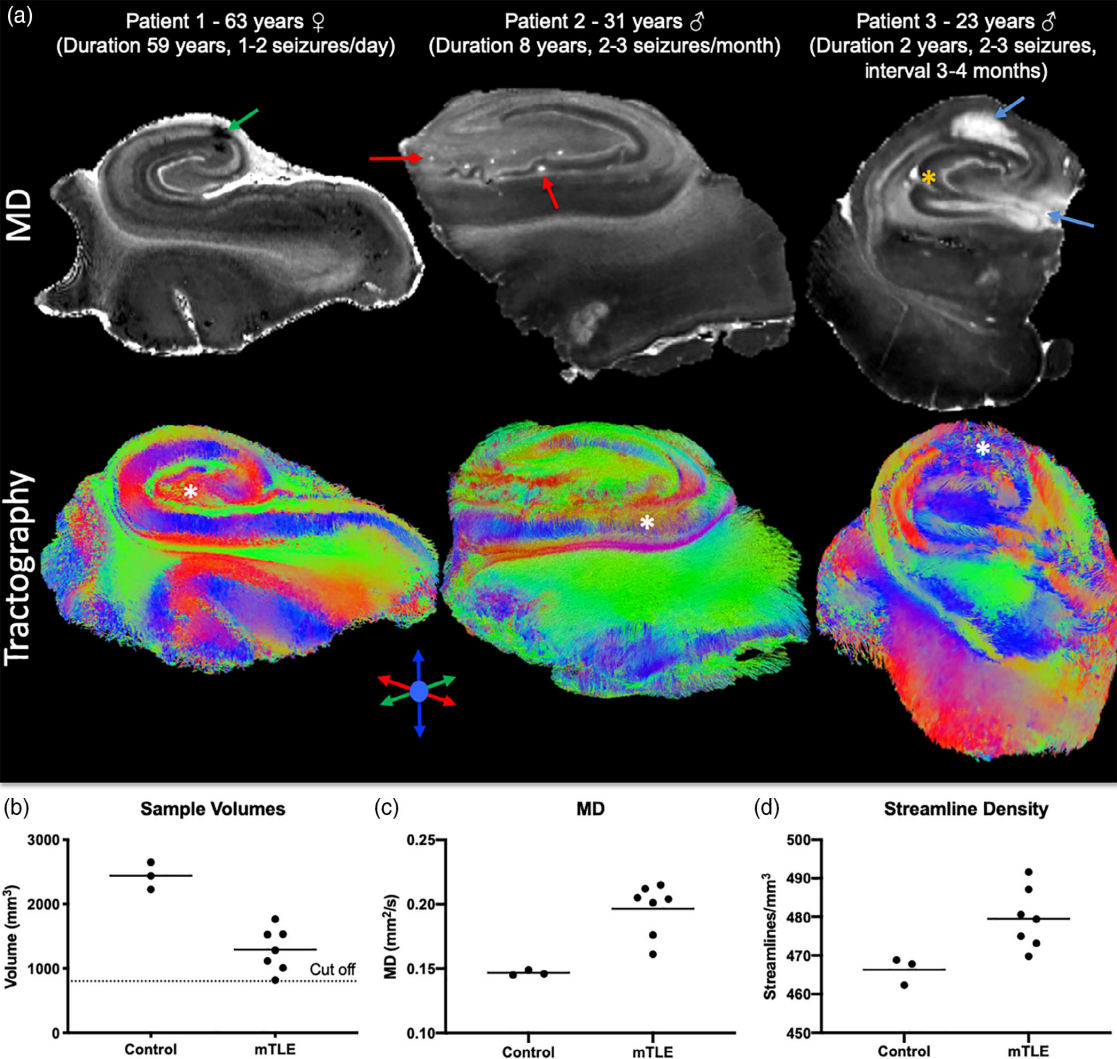
Diffusion MR imaging of control and epileptic hippocampi. (a) MR imaging of hippocampal samples excised from patients with intractable epilepsy poses several challenges. Typically, only the tail region of the excised sample was available for the imaging study, hence limiting the regional evaluation of pathology. Surgical manipulation can also induced injury that leads to small bleeds (green arrow). These hypointense spots are excluded from defining a region of interest. In other cases, blood vessels can produce a hyperintense signal that reflects the presence of freely moving liquid through these (red arrows). Epileptic samples can also contain different levels and types of pathology. A hyperintense region can indicate surgical trauma, but can also be due to reactive gliosis (blue arrows). Anatomical malformations can also be evident (orange asterix). (b) As only the posterior body or tail is typically available for imaging studies, the sample size was approximately a third of the control samples (individual data points represent each subject, line reflects the group mean). For this reason, in control samples only the posterior body and tail region was included for analysis. Samples with a volume of <800 mm^3^ were excluded from the study, as this portion of the tail typically only afforded a delineation of the dentate gyrus and did not allow a robust comparison between controls and patients. (c) The mean diffusivity (MD) of the patients' samples was higher than controls. (d) The streamline density in epileptic samples was denser than in controls with a mean difference of 13 streamlines/mm^3^

The control subjects' hippocampi provide anatomical context for samples excised from patients with intractable epilepsy. *En bloc* epileptic hippocampi resections can be anatomically very similar to control hippocampi, but can also have artifacts due to sample excision (i.e., surgical trauma), fixation, sclerosis, anatomical malformations and aberrant connectivity (Figure 3a). As only the posterior body region was typically available for ex vivo imaging, the overall sample size of the epileptic hippocampi was lower (Figure 3b). Epileptic samples revealed a 26% greater diffusivity (Figure 3c) with on average 23 more streamlines/mm^3^ than in control post‐mortem samples (Figure 3d).

### 3.3 Hippocampal layer diffusion properties and the impact of epilepsy

**FIGURE 4:**
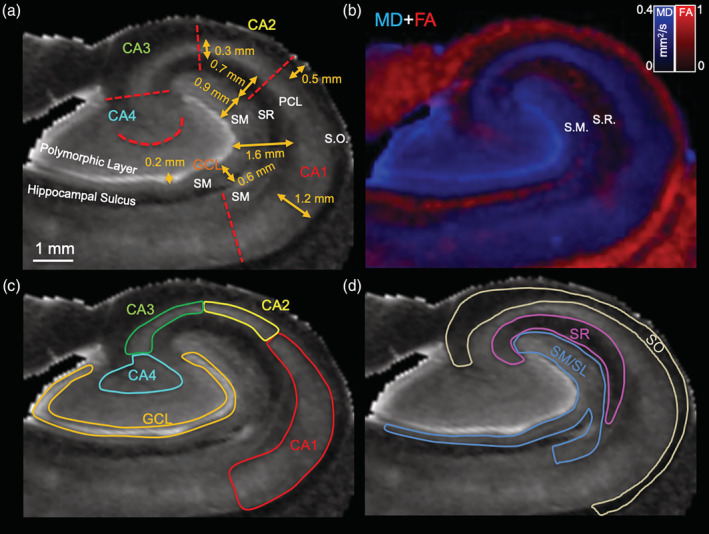
Defining regions of interest (ROIs) in human hippocampi. (a) Delineation of anatomical landmarks on mean diffusion (MD) images is achieved based on signal contrast. The granule cell layer (GCL) of the dentate gyrus (DG) is very hyperintense and affords easy delineation. This segementation further defines the stratum moleculare (SM) and the polymorphic cell layer of the hilus. The hippocampal sulcus further divides the S.M. The stratum radiatum (SR) is hypointense and can be separate from the more isointense SM and the pyramidal cell layer (PCL). The PCL can be divided in subfields defining cornu ammonis (CA) 1–4. Size measurements highlight the need for a high resolution with the GCL measuring merely 0.2 mm, that is, two voxels. (b) An overlay between color‐coded MD (blue) and fractional anistropic (red) images can further aid in refining individual cell layers as contrast is more clearly defined for some regions (e.g., SR). (c) CA1‐4 subfields were defined based on anatomical markers. Specifically, the end of the hippocampal sulcus defined the start of CA1. The start of CA2 was specified at the half way point of the external limb of the GCL. The start of CA3 coincided with the apex of the PCL and extended to the end of the external limb of the GCL. CA4 extended from this point into the hilus, but was contrasted with the polymorphic layer that was more hyperintense. The polymorphic layer and GCL in addition to the SM along the hippocampal sulcus were defined as the DG. (d) The stratum oriens (SO) was defined as the thin hypointense layer overlaying the PCL along the CA1‐CA2 regions. Overlaying this thin layer was the more isointense alveus that extended into the subiculum. The SR was a more hypointense region adjacent to the PCL. The SM was more isointense than the SR. The stratum lacunare (SL) was not robustly distinguished and is considered to be part of the region defined as SM here

Mesoscale images afford the visualization of individual cell layers to analyze how epilepsy affects their volume and connectivity. A 0.1 mm isotropic resolution is required to reliably delineate, for instance the GCL, which is only ~0.2 mm thick (Figure 4a). Sizing of individual layers is important to achieve an appropriate resolution to delineate these structures and define ROIs for seed regions. Overlaid color‐coded MD and FA images help to refine and resolve boundaries between lamina, such as the SM and SR along the external limb of the GCL (Figure 4b). Hippocampal subfields (CA1‐4) can be defined for the PCL and the GCL of the DG (Figure 4c). A combined SM/SL, as well as SR and SO ROIs were robustly differentiated from the PCL and GCL (Figure 4d).

**FIGURE 5:**
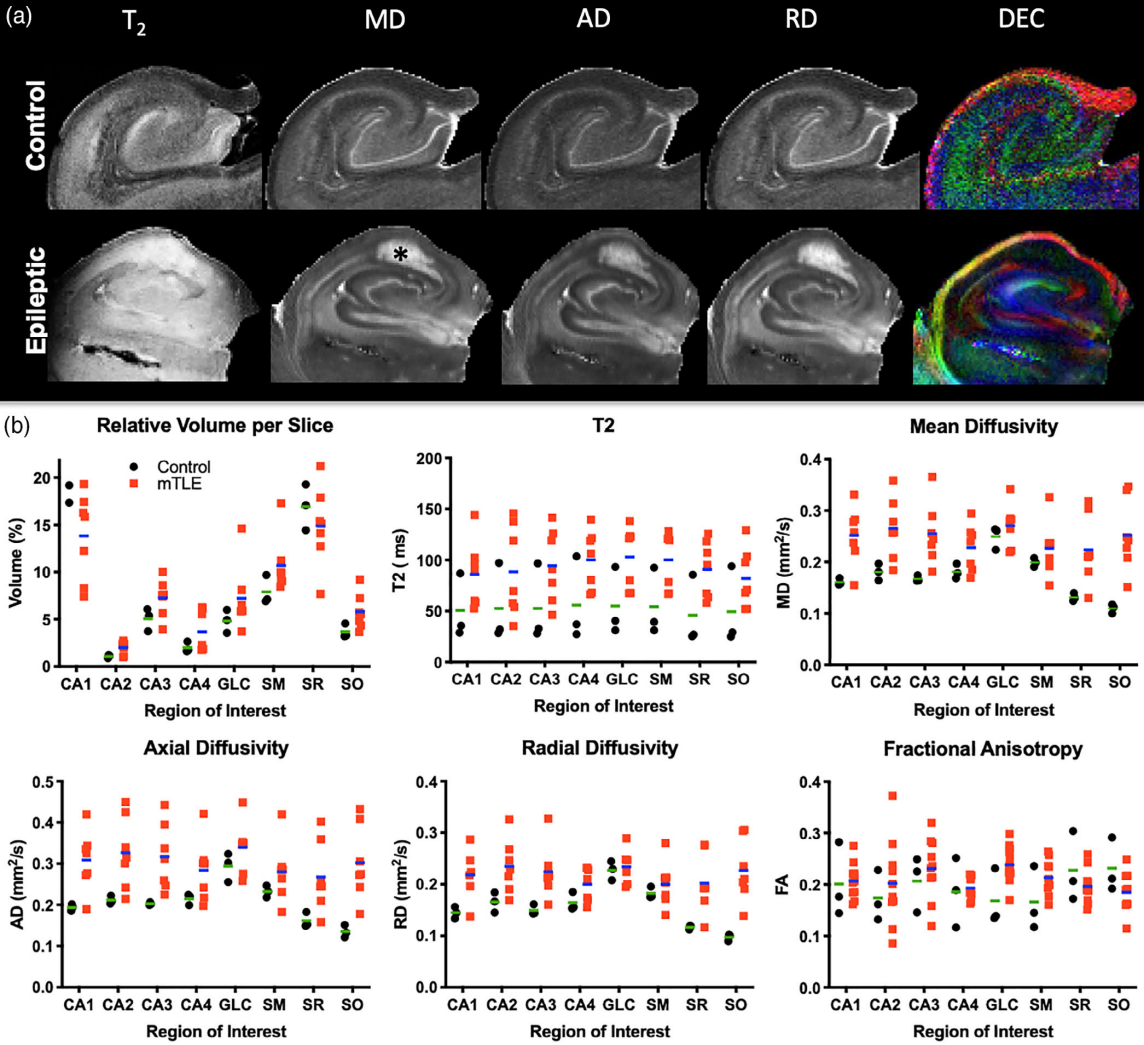
Comparisons of regional volumes and scalar indices. (a) Coronal MR images and scalar maps of a control and epileptic hippocampus. In the epileptic hippocampus a hyperintense region (*) is evident in all scans that is due to tissue damage and reactive gliosis. The diffusion encoded color (DEC) image reflects direction of primary diffusion within a voxel encoded by color. (b) Regional volume, T_2_ signal intensity, mean diffusivity (MD), axial diffusivity (AD), radial diffusivity (RD) and fractional anisotropy (FA). Variability of measurements on FA were equivalent between groups, but variability in MD, AD, and RD was much higher in epileptic samples (individual data points represent each subject, line reflects the group mean)

To evaluate the impact of epilepsy on the diffusion properties of individual hippocampal layers, ROIs were compared for T_2_ signal intensity, MD, AD, RD and FA (Figure 5a). To account for differences in samples size, the relative volume of ROIs was calculated by accounting for the number of slices in each sample to provide a normalized measure to illustrate differences in regional volume, T_2_, MD, AD, RD and FA (Figure 5b). It was evident that epilepsy samples were more variable on these measures compared to controls. Signal intensity measures overall were higher in epilepsy samples, potentially reflecting the difference in fixation between both types of samples.

### 3.4 Tractography reveals increased connectivity in samples from epilepsy patients

**FIGURE 6:**
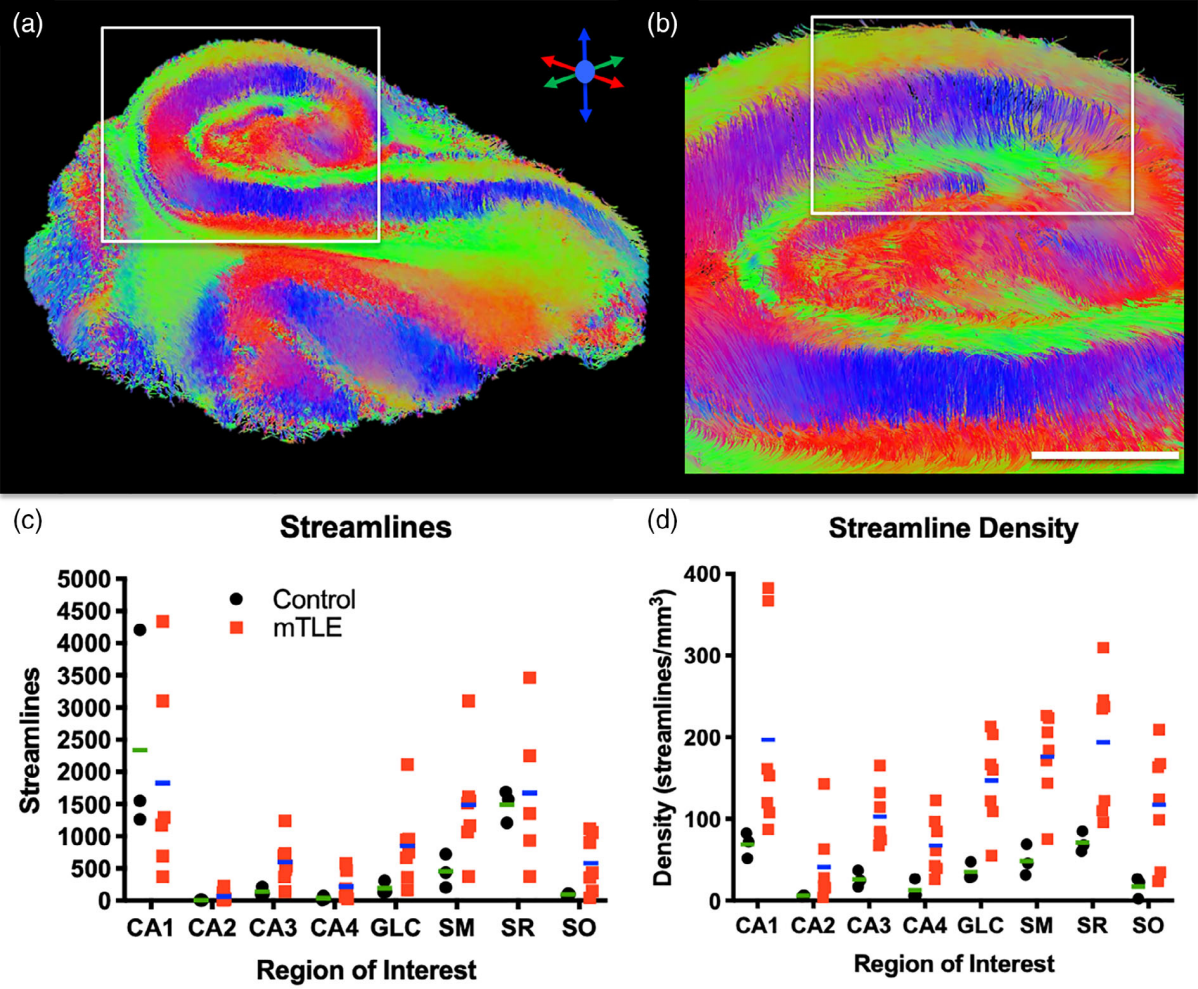
Hippocampal connectivity. (a) Tractography on the hippocampal samples revealed fiber tracts connecting different regions. (b) Perpendicular streamlines emanating from the pyramidal cell layer form connections with deeper seated layers. (c) Streamlines from seeds in different regions indicated more fibers in epileptic samples compared to controls (individual data points represent each subject, line reflects the group mean). Variability in epileptic samples was higher compared to controls. (d) To account for potential differences in regional volumes between both experimental groups, streamline density was calculated. CA1, SM and SR exhibited a higher streamline density in cases with epilepsy

To investigate alterations in hippocampal connectivity in patients with mTLE, tractography was performed to visualize streamlines representing neuronal connections. Streamlines are generated throughout the epileptic hippocampus and reveal a unique insight into its connectivity (Figure 6a). For instance, vertical fibers, defining the Schaeffer collaterals of CA1, are visible and interface with horizontal running fibers through the SR (Figure 6b). The streamlines of the hippocampus reflect the laminar organization and provide a means to assess connectivity that is not afforded by scalar images. The total number of streamlines in the epilepsy samples was very variable (Figure 6c). In part, this is a reflection of different sample volumes. The CA1 region produced the most streamlines, whereas CA2 produced the fewest streamlines. To account for volumetric differences, streamline density was calculated (Figure 6d). The streamline density for mTLE samples were more variable than for controls, reflecting the impact of disease state on connectivity measures. Epilepsy samples in all regions had a higher streamline density compared to controls. This difference is conceivably due to a better tissue quality of epilepsy samples, as these were fixed straight upon excision and did not undergo post‐mortem decay, but some of these differences could also be the result of the disease state of the tissue. The fundamental difference in the timing of tissue fixation here does not afford a distinction of these two possibilities. Nevertheless, the control samples provide anatomical context regarding regional differences of streamline densities.

**FIGURE 7:**
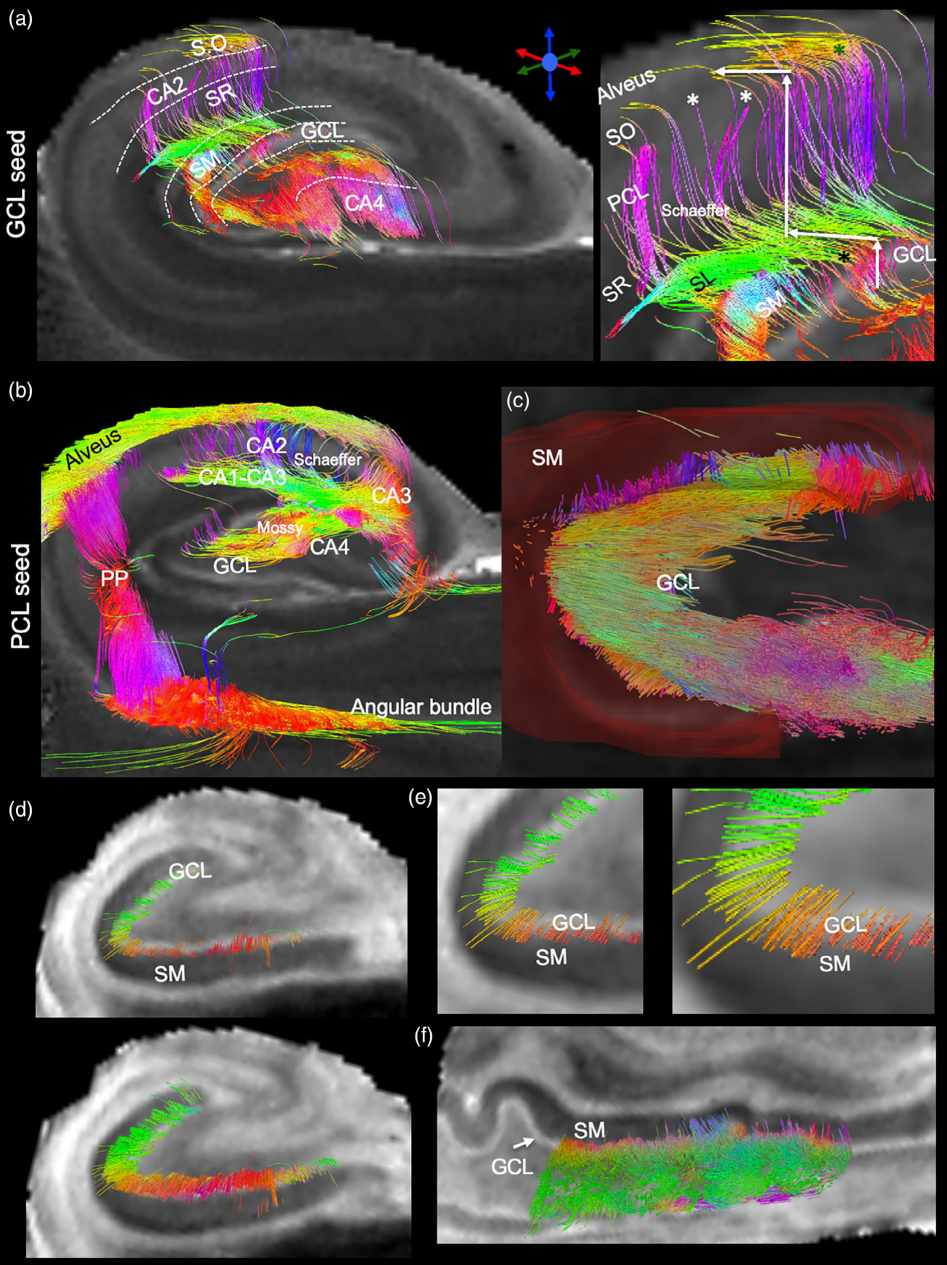
Regional connectivity. (a) A seed in the granule cell layer (GCL) revealed connections to the CA4 pyramidal cell layer (PCL), as well as to the CA2. Streamlines to CA2 span connections between multiple cell layers. Although some connections terminated within the appropriate cell layer (white *), tracing did not consistently terminate in the stratum moleculare (SM) (*), or PCL (*). (b) Seeding in the PCL uncovered an extensive network of connections. Notably, the performant path (PP), alveus, CA1‐CA3 connections, Schaeffer collaterals of the PCL, as well as GCL to CA4 (Mossy fibers) and CA3 projections. (c) Axonal projections from the the GCL formed aberrant connections that terminated in the SM (region defined by semi‐transparent red outline) and could produce reverberant excitatory networks that drive seizure activity. (d) A closer visualziation of streamlines across at different slice positions with greater contrast between the GCL (seed) and SM (terminal region) further highlight individual streamlines crossing from the GCL to the SM in a perpendicular direction to the GCL. (e) Higher magnification images demontrate how streamlines from the GCL fan into the SM. Most streamlines are short connections that terminate early in the SM, but a few penetrate deeper into the cell layer. Deeper penetration are mostly at the arch of the GCL rather than in the internal or external limb. (f) A sagittal view of the GCL‐SM connectivity indicates that these streamlines cover the entire length of these layers, rather than being confined to a small portion

**FIGURE 8:**
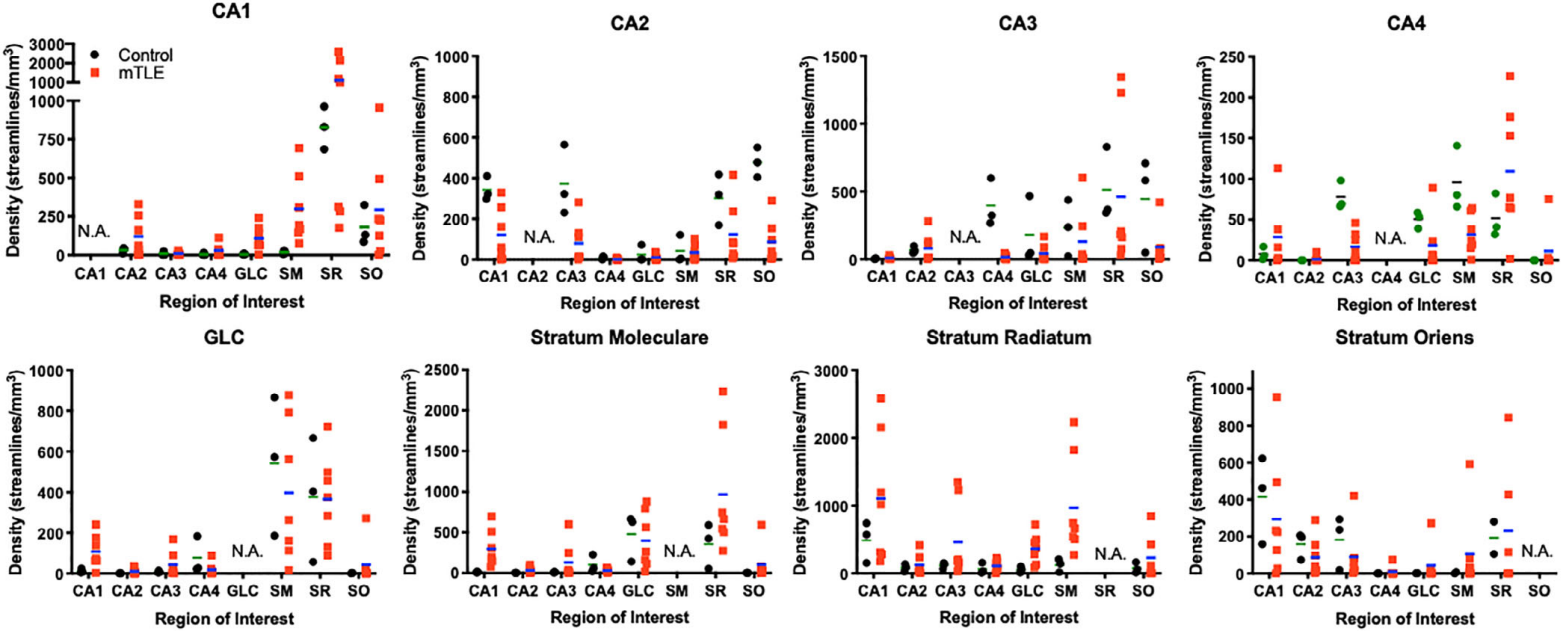
Quantitating regional connectivity. Comparisons between control and epileptic samples revealed a systems view of hippocampal connectivity between seed and terminative regions of interest (individual data points represent each subject, line reflects the group mean)

To evaluate regional connectivity, streamlines generated from a seed in one region and terminating in another were compared. The GCL seed, for instance, produced streamlines that reflect GCL‐CA4, but also GCL‐CA2 connectivity (Figure 7a). The GCL‐CA2 streamlines indicate that “multisynaptic tracing” of an entire pathway can occur (i.e., axonal connections between >2 cell layers), rather than definition of a single connection between two regions. Often this is reflected in a change of streamline direction, as illustrated here with streamlines connecting GCL to the SO across multiple slices. The appearance of these streamlines is distinct from streamlines that pass through a single cell layer, which do not have an abrupt change in direction. A PCL seed for CA1‐CA4 revealed streamlines of a very extensive intra‐hippocampal network (Figure 7b), including connections to CA1, CA2, CA4 and the GCL. Streamlines further connected through the alveus and fed into the perforant path to reveal extra‐hippocampal connectivity through the angular bundle. To specifically investigate the hypothesized “aberrant” connectivity between the GCL and SM in epilepsy, streamlines were evaluated in relation to the ROI for the SM (Figure 7c). An extensive number of streamlines terminating in the SM were evident (Figure 7d). Higher magnification images reveal the perpendicular nature of streamlines emanating from the GCL to terminate predominantly in the adjacent inner rim of the SM (Figure 7e). A few streamlines in the arch of the GCL, however, showed a deeper pentration of the SM. This streamline pattern was found throughout these two structures (Figure 7f). Evidence of these streamlines was also found in controls. Quantitation of streamlines connecting one region with another afforded a comparison between control and mTLE samples (Figure 8). Although control samples did not produce much variability in measurements, there was considerable variability in mTLE patients that is potentially reflective of the length or disease burden.

### 3.5 Fractional anisotropy in stratum radiatum correlates with seizure frequency

**FIGURE 9:**
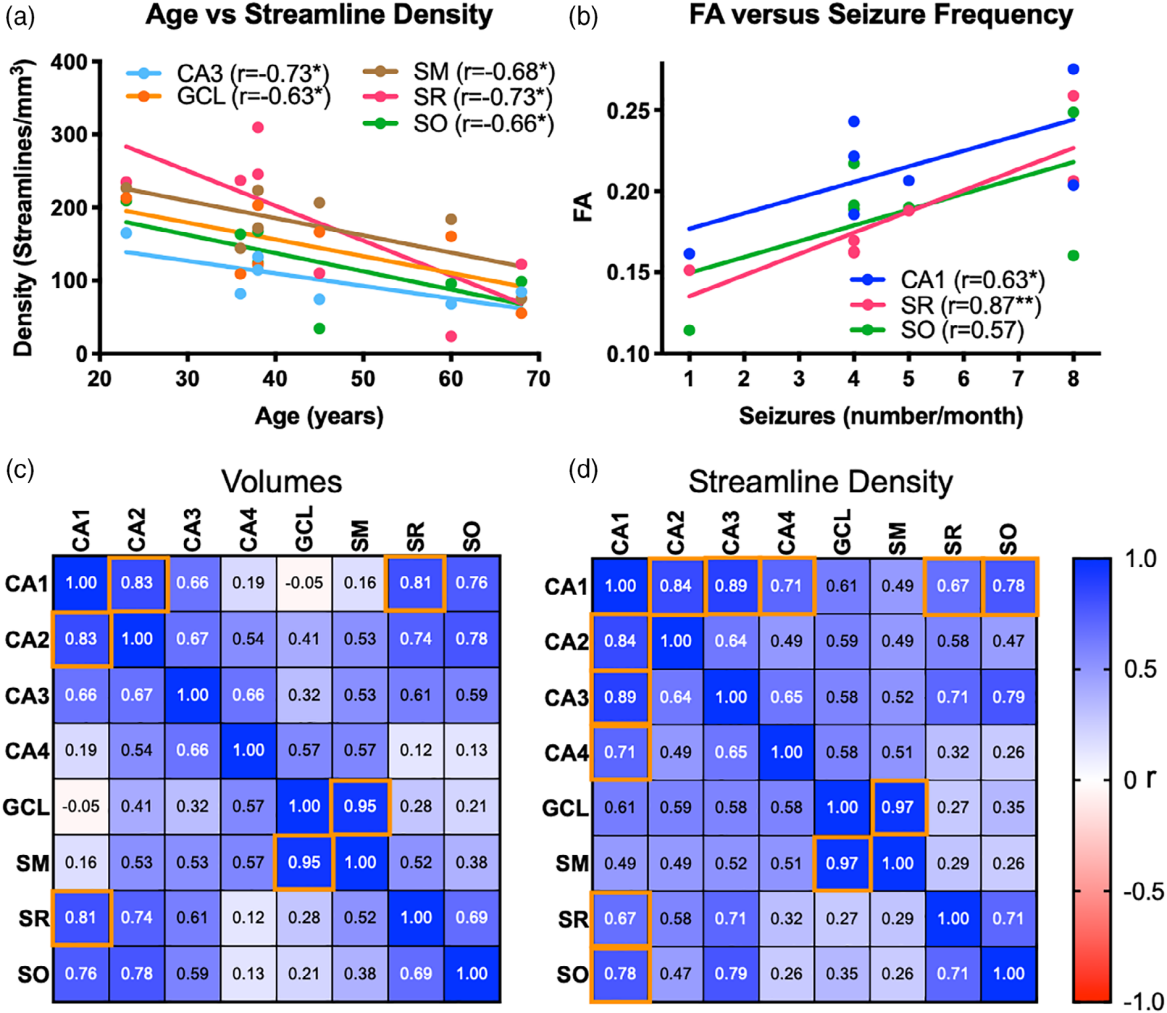
Correlations between disease burden and MRI measures. (a) Age is negatively correlated (*p < .05) with streamline density (individual data points represent each subject). Older subjects have reduced axonal projections in CA3, GCL, SM, SR and SO. Age is therefore a major co‐variate with older subjects. (b) The severity of epilepsy, as indicated by seizure frequency, correlated highly with FA, particularly in the SR,as well as CA1 and SO (*p < .05; **p < .01). (c) The correlation matrix detailing *r* values between ROIs and volume uses a colorimetric scale to indicate the strength and direction of correlation (warm to cold). The strongest correlation (*r* = 0.95) was found between GCL and SM, but strong correlations were also evident for CA1‐CA2, CA1‐SR, CA1‐SO, CA2‐SR and CA2‐SO. Weaker, but significant, correlations were also evident for CA3 with CA1, CA2 and CA4. (*r* values in white *p* < .05, FDR in orange square *q* < 0.05). (d) The volumetric correlations also translate into correlations of streamline density between these ROIs with GCL being strongly correlated with SM. CA1 and CA2 streamline density was also strongly correlated with SR and SO. CA1 and CA2 subfields were also highly correlated with each other, but showed a weaker association with CA3. The pattern of streamline density in CA3 and CA4 were also significantly correlated

To determine if variability of mTLE samples is potentially a reflection of patient characteristics (i.e., age, disease severity, length of the disease), correlational analyses were conducted. There was no correlation between disease severity and its length (*r* = −0.19, n.s.) or patient's age (*r* = −0.03, n.s.). However, the length of the disease was correlated with the patient's age (*r* = 0.61, *p* < .05), indicating that older patients suffered for longer from mTLE. The patient's age was also significantly correlated with the volume of CA1 and CA3 (Table 2). The streamline density in CA3, GCL, SM, SR and SO were correlated with age (Figure 9a), suggesting that the patient's age is an important co‐variate in these measures. Older subjects generally had a lower streamline density and smaller regional volumes. A reduction in CA3 and CA4 volumes was correlated with the length patients suffered from mTLE (Table 2). Streamline density in CA4 was also negatively correlated with disease duration. A reduced streamline density was observed in CA1 and CA3 with an increased seizure frequency, indicating that disease severity impacted hippocampal connectivity (Table 2). In contrast, age and disease duration did not significantly correlate with scalar measurements in different ROIs (Table 3). Only disease severity correlated with scalar measurements. FA values in CA1 and SR positively correlated with disease severity (Figure 9b). FA in SR was very strongly correlated (*r* = 0.87, *p* < .05) with disease severity. Severity also correlated with MD and RD values in CA3 and MD, AD and RD values in CA4 (Table 3).

**TABLE 2:** Correlations coefficients of MR volumes and streamline density with disease status

Region	MRI	Age	Length	Severity
CA1	Volume	−0.66*	−0.38	0.41
	Density	−0.59	−0.42	−0.63*
CA2	Volume	−0.48	−0.46	0.22
	Density	−0.30	−0.14	−0.46
CA3	Volume	−0.81*	−0.91**	0.41
	Density	−0.73*	−0.56	−0.70*
CA4	Volume	−0.30	−0.74*	0.45
	Density	−0.52	−0.72*	−0.34
GCL	Volume	−0.13	−0.25	0.04
	Density	−0.63*	−0.11	−0.42
SM	Volume	−0.30	−0.43	0.02
	Density	−0.68*	−0.14	−0.20
SR	Volume	−0.48	−0.34	0.06
	Density	−0.73*	−0.55	−0.11
SO	Volume	−0.52	−0.39	0.27
	Density	−0.66*	−0.22	−0.55

* *p* < .05.

** *p* < .01.

**TABLE 3:** Correlation coefficients of MR scalar indices with disease status

Region	MRI	Age	Length	Severity
CA1	FA	0.34	0.31	0.63*
	MD	0.01	−0.22	0.43
	AD	0.05	−0.17	0.43
	RD	−0.02	−0.25	0.43
CA2	FA	0.27	0.40	0.15
	MD	−0.41	−0.38	0.34
	AD	−0.28	−0.20	0.20
	RD	−0.46	−0.47	0.40
CA3	FA	0.16	0.54	0.30
	MD	−0.35	−0.36	0.63*
	AD	−0.28	−0.21	0.59
	RD	−0.38	−0.46	0.63*
CA4	FA	−0.12	−0.36	0.38
	MD	−0.36	−0.25	0.68*
	AD	−0.32	−0.30	0.65*
	RD	−0.38	−0.21	0.67*
GCL	FA	−0.05	−0.27	−0.31
	MD	−0.14	−0.32	0.22
	AD	−0.13	−0.13	0.42
	RD	−0.15	−0.07	0.58
SM	FA	0.04	−0.03	−0.09
	MD	−0.15	−0.42	0.26
	AD	−0.16	−0.27	0.41
	RD	−0.21	−0.30	0.51
SR	FA	−0.46	−0.46	0.87*
	MD	0.10	−0.33	0.26
	AD	−0.13	−0.37	0.60
	RD	0.25	−0.13	0.09
SO	FA	0.54	0.10	0.57
	MD	−0.12	0.01	0.27
	AD	0.03	−0.04	0.27
	RD	−0.12	−0.05	0.19

* *p* < .05.

To determine if changes in one region are associated with changes in another, volume and streamline density were correlated between ROIs. Strong correlations for volumetric change were evident in closely associated regions, such as CA1 and CA2, as well as CA1 with SR and SO (Figure 9c). However, the more distant regions, such as CA4, GCL and SM were not correlated with volume in CA1. The strongest volumetric correlation was found between GCL and the adjacent SM (*r* = 0.95, *p* < .0001, *q* < 0.05). No negative correlations were evident, where one region's enlargement was associated with another's atrophy. This pattern of correlation was mostly mirrored by streamline densities between regions (Figure 9d), with the key exception that CA1 and CA3 were more highly correlated than CA1 and CA2. Streamline density in the GCL and SM region also exhibited the highest correlation (*r* = 0.97, *p* < .0001, *q* < 0.05).

## 4 DISCUSSION

A major limitation to our understanding of mTLE is a lack of imaging tools that can visualize connectivity in the temporal lobe. We here demonstrate that mesoscale diffusion MR imaging of excised tissue samples can provide unique insights into the seizure networks associated with mTLE. Specifically, we here demonstrated: (1) a robust identification and measurement of hippocampal layers at the mesoscale, affording a comparison at the individual cell layer level; (2) diffusion MR and streamline density revealed microstructural changes that distinguish control and mTLE samples; (3) regional connectivity changes, especially in the PCL of subfields CA2 and CA4 are evident in mTLE patients; (4) age is a major covariate in hippocampal streamline density; (5) streamline density in CA1 and CA3 is negatively correlated with seizure severity; and (6) seizure frequency is also correlated with diffusion changes in CA3, CA4 and SR. As FA variability in SR was very strongly correlated with seizure frequency, this measurement may implicate the SR in mediating seizure activity. These results also indicate that mesoscale imaging of surgical samples excised from mTLE patients can provide a unique systems view of hippocampal connectivity that cannot be gained from histological studies.

### 4.1 Mesoscale diffusion MR imaging of hippocampal connectivity in human tissue samples

The hippocampus is a major focus of mTLE, with atrophy being one of the hallmarks of the disorder and a selection criteria for surgical resection (Farid et al., 2012; Theodore et al., 1999), Hippocampal atrophy is also associated with Alzheimer's disease (Ten Kate et al., 2017) and depression (Stratmann et al., 2014), indicating a wider implication of studying hippocampal connectivity and the impact of different neurological conditions (Bartsch, 2012). Age is a major factor determining hippocampal volumes (Mueller et al., 2007; Wolf, Fischer, de Flores, Chetelat, & Fellgiebel, 2015) and as indicated here is a potential co‐variate with a patient population spanning a wide age range, including patients with mTLE. Age‐related changes in hippocampal microstructure have mainly been associated with the CA1 region in the body and tail (Wolf et al., 2015). Regional volumetric changes in the head, for instance, could hence potentially provide defining characteristics to differentiate aging and disease, but regional changes in mTLE patients are poorly understood. mTLE patients have been reported to exhibit a significant volume loss in hippocampal subfield CA1 to CA4, as well as the dentate gyrus, subiciulum and fimbria (Schoene‐Bake et al., 2014), but little is known about how differentially these changes affect the tail, body or head regions. Subfield pathology in mTLE indicates that there is greater pyramidal cell loss in CA1 compared to CA2, CA3 and CA4 (Steve, Jirsch, & Gross, 2014). A reduced neuron count in CA3 was also evident in relation to CA2, whereas neurons in CA2 are mostly spared (Schoene‐Bake et al., 2014). It has further been suggested that hippocampal sclerosis (HS) type 1 mTLE subtype has a lower CA4‐DG volume on MRI and neuronal density on histology compared to HS2 (Peixoto‐Santos et al., 2018). Interestingly, cellular loss in CA3, CA4 and dentate gyrus in patients with mTLE were associated with a decline in declarative memory, but loss of cells in CA1 was not (Coras, Pauli, et al., 2014). Nevertheless, no significant relationship between in vivo MRI‐based hippocampal subfield volumes and clinical variables (i.e., duration and age of onset) have been found (Bower, Kilpatrick, Vogrin, Morris, & Cook, 2000; Kreilkamp, Weber, Elkommos, Richardson, & Keller, 2018).

Although hippocampal subfields are assigned to in vivo MR images using an automated (Sone et al., 2016) or manual segementation protocol (Peixoto‐Santos et al., 2018), the spatial resolution achieved is currently insufficient to identify the PCL in subfields CA1‐CA4 or the GCL in the DG (Modo et al., 2016). Nevertheless, histological studies on neuronal loss focus on these specific cell layers (Steve et al., 2014). This discrepancy in resolution can potentially explain the lack of correlation between MR measures and clinical variables. To overcome this issue, several studies have used ex vivo hippocampus samples to achieve a high in‐plane spatial resolution to visualize individual cell layers (Chakeres et al., 2005; Coras, Milesi, et al., 2014; Yushkevich et al., 2009), but typically these studies used a disproportionate slice thickness that limits volumetric and tractographic analyses. As demonstrated in our study, measurement of volumetric changes in these cell layers can only be achieved with at least a 0.1 mm isotropic resolution. This mesoscale resolution is required to sufficiently resolve, for instance, the GCL of the DG, which measures just 0.2 mm across, and separates the surrounding polymorphic layer and the SM. Minimizing partial volume effects of cell layers, such as the GCL, is therefore quintessential to ensure a robust tractography that identifies the diffusion directions within each voxel to serve as seeds, as well as to define termination values for individual layers (e.g., FA value).

Mesoscale MR image segementation of individual cell layers and their classification into CA1‐CA4 subfields here revealed a volumetric difference in the PCL of CA1 between controls and mTLE patients, akin to observations based on histological analyses (Steve et al., 2014). Due to partial volume effects of neighboring SM and SO, the low resolution of in vivo subfields measurements lacks the specificity and sensitvity to account for the neuronal loss in the PCL. A higher spatial resolution is therefore required to increase the diagnostic utility of subfield measurements. Differences in diffusion properties between cell layers also advocates for the use of a higher spatial resolution to improve the diagnostic value of these scans. At a mesoscale resolution, diffusion MR images afford the robust identification of individual layers based on signal intensities in MD, RD and AD, as well as based on FA maps (Ly et al., 2020). Specifically, MD, a measure of cellularity, was consistently lower in mTLE patients, as one would expect due to neuronal loss in the PCL of CA1, for instance. However, SO overlying the PCL of the CA1 subfield was the only region showing a specific regional difference between mTLE patients and controls. Although RD and AD produced the same effects, FA did not reveal any significant difference between controls and mTLE patients. mTLE patients exhibited a large variance compared to controls, potentially reflecting the variability in clinical measures.

### 4.2 MR imaging biomarkers and connectivity in mTLE

The variability of FA in the SR was dependent on seizure severity, but was not affected by length of the disease or the age of the patient. FA in CA1 was negatively correlated with the number of seizures that patients experienced, but exhibited a lower level of association than the SR region. A strong correlation between an imaging measure and clinical variable indicates that FA in SR and CA1 is indicative of pathological changes in these regions that are important in seizure activity. We further found that MD and RD in the CA3 and CA4 region correlated with disease severity. Streamline density in CA1 and CA3 also correlated with seizure frequency, highlighting the importance of these regions to the seizure network. Interestingly no correlation of the GLC with clinical variables was evident. Changes in clinical variables over time were not captured here and could provide another dimension of measurement that can contribute to anatomical changes Consequently, it remains unclear if these changes are a cause or a consequence of disease severity. Striving to achieve a mesoscale resolution in vivo may be a worthwhile endeavor as a diagnostic tool in epilepsy, but it will also provide new insights into the networks that underlie seizure activity.

Interestingly, streamline density of mTLE patients in CA1, SM and SR was higher than in controls. Streamline densities between regions produced the most differences between controls and mTLE patients. Still, it is unclear if connectivity changes are upstream or downstream of the seizure generating region. It is also conceivable that there is no specific individual locus generating seizures, but that it is an extended change in connectivity throughout the hippocampus that is driving a reverberant activity. Is a loss or increase in connectivity producing a seizure network or is this an adaptive change to neuronal loss in a specific region? Comparison between subjects with a short history of epilepsy versus those with a prolonged disease history in a future study could address which network changes are associated with seizure generation, rather than adaptive changes. It is noteworthy that overall, streamline density was increased without a region having undergone a decrease in apparent connectivity. Loss of connectivity is therefore unlikely to be a factor in seizure generation. However, these increases in streamline density are potentially a function of a reduced regional volume. Similar observations of an increased “connectivity” have been reported for subfields in vivo in patients with mTLE (Rutland et al., 2018), but establishing an absolute change in connectivity would require a whole sample comparison between controls and mTLE patients. Inclusion of whole hippocampal samples of mTLE patients would improve this analysis, as well as determine if different parts of the hippocampus are more affected by the disease than others. However, differences in tissue fixation poses challenges for quantitative comparisons between ex vivo control and epilepsy samples.

A hypothesized aberrant connection between the DG and SM is thought to produce an excitatory reverberant network that sustains seizure activity (Blumcke & Spreafico, 2012; Houser, Zhang, Peng, Huang, & Cetina, 2012). In humans, this is supported by evidence of excitatory glutamatergic axons in the inner SM, which are not present in healthy controls (Frotscher, Jonas, & Sloviter, 2006; Proper et al., 2000; Sutula & Dudek, 2007). In animal models, histological tracing methods have demonstrated that these aberrant axons are derived from granule cells in the dentate gyrus (i.e., Mossy fibers), but these methods cannot be applied to human tissue samples to directly trace axons between histological slices. Tractography can be used as an alternative method to investigate this type of connectivity in surgical samples from patients with mTLE (Ly et al., 2020; Modo et al., 2016). Although we here detected streamlines spanning between the GCL and SM regions in mTLE samples, control samples also revealed some streamlines between these two regions. In both cases, these were mostly terminating in the inner region of the SM, as documented in histological studies of mTLE patients (Buckmaster, 2012). Although a minor mossy fiber projection into the granule cell layer has been demonstrated using the Timm stain (Haug, 1974), the presence of these streamlines in controls nevertheless questions the “aberrant” nature of these. It is conceivable that glutamatergic axons are aberrant in mTLE, with no glutamatergic axons in the healthy hippocampus (Proper et al., 2000). Another type of axon might hence provide an underlying connectivity between these two structures that is not readily identified using histology. A more likely consideration is that better definitions of individual ROIs are required to conduct more precise tractography, potentially requiring an even higher spatial resolution. The mossy fiber pathway from the GCL to the SM in CA3 is known to connect with pyramidal cells in the SL (Frotscher et al., 2006). This could be the basis of the streamlines observed here terminating inappropriately. Our inability here to identify the SL could lead to the tracing of connections that terminate in this layer, rather than the SM. Further divisions of SM, SR, SO according to subfields CA1‐CA4 might also further refine pathway separations.

### 4.3 Limitations of ex vivo tissue sample imaging

It is worth noting that tractography in gray matter produces several challenges to generate streamlines that are not currently addressed using tractography paradigms used in white matter tracing. Firstly, FA values are much lower and more homogenous within gray matter than between white and gray matter. An FA threshold is typically used to define termination values for white matter tracts. FA therefore has a more limited utility to define the termination of fiber tracings in indivudal cell layers. Secondly, the use of only 12 diffusion directions limits tractography paradigms to account for the angular resolution of fibers, fanning, as well as crossing or kissing fibers, which are common occurances in gray matter. We therefore recommend the use of more sophisticated diffusion imaging acquisition strategies that can acquire a higher order of diffusion encoding directions and multiple b‐values (Shi & Toga, 2017). However, to acquire these in a reasonable time frame, more developments in acquisition strategies are required. For instance, implementation of compressed sensing can accelerate acquisition by a factor of 8 (Wang et al., 2018; Zhang et al., 2020). Thirdly, better methods are required to distinguish single connections from tracing through multiple cell layers. For instance, we here visualized the GCL‐PCL pathway across multiple cell layers and potentially could define each single aspect of the connection by using different angular thresholds, maximal distance and subtle differences in FA to terminate tracing in a single cell layer. This indicates that potentially each “connection” would require separate tractography settings. Tractography paradigms therefore need to be specifically developed for these circumstances.

Knowledge of normative connectivity in healthy subjects is important to define the impact of disease on connectivity. At present, no normative data for the human hippocampus is available that would define appropriate connections between ROIs. A major challenge in developing such an atlas is tissue availability from control subjects. Control hippocampi are only available from cadavric tissues. Tissue decay and cellular loss, especially pyramidal cells of CA1 due to delays in fixation, can be a concern regarding the quality of these samples (D'Arceuil & de Crespigny, 2007; Scheurer et al., 2011; Shepherd et al., 2009). In contrast, mTLE samples are typically fixed immediately upon excision and hence produce a higher quality of tissue preservation. For instance, lower T_2_ and MD values in controls here are a potential reflection of this difference in sample preparation. A recent study indicated that ex vivo imaging of hippocampi from mTLE patients reflected the anatomy and tissue properties of in vivo scans (Wisse et al., 2017). Ex vivo imaging of mTLE hippocampi therefore is likely to be a valid representation of hippocampal connectivity in vivo. The generation of a normative connectivity from control samples for comparison might be the main challenge to contextualize the variability in connectivity observed in mTLE patients. Preclinical studies in animal models of epilepsy can potentially bridge this knowledge gap by comparing high quality control and epileptic hippocampi (Reddy et al., 2019; Shepherd, Ozarslan, King, Mareci, & Blackband, 2006).

A further issue with comparisons between mTLE patients and controls is the challenge to account for age. As we have shown here, age is an important variable in the volumetric analysis of hippocampal regions, as well as in streamline density. However, it was not associated with clinical variables or scalar measurements. Differences in FA in the SR and its correlation with seizure severity is hence independent of age. A widespread age range here afforded a correlation analysis to yield significant results even with a small sample size. Sufficient power was achieved for very strong correlations, but medium‐sized correlations did not yield significance and would require larger sample sizes to reveal their utility and contribution to a seizure network. Direct comparison of patients with a short or long course of the disease would also be advantageous to distinguish adaptive from putative causal changes in connectivity. In the context of diagnostic radiology, inclusion and differentiation of multiple types of epilepsy, such as HS1 versus HS2, would afford an evaluation of how these imaging characteristics could inform a differential diagnosis (Peixoto‐Santos et al., 2018) and bridge the gap between radiological and neuropathological tools.

## 5 CONCLUSION

Understanding connectivity changes underpinning epileptic activity of the hippocampus remains a major challenge. We here demonstrated that mesoscale diffusion MR imaging of excised hippocampal samples from patients with mTLE can provide novel insights into microstructural changes in individual cell layers, as well as their connections. Correlations between these MR measures and clinical variables revealed several changes that could potentially provide a means to establish which localized pathological changes are relevant to seizure activity. Of these, FA values in SR produced the strongest correlation with seizure frequency. Dramatic improvements in spatial resolution are required for diagnostic imaging to identify individual cell layers and to measure connectivity. As demonstrated here, ex vivo hippocampal samples can provide a useful tool to define novel targets for diagnostic imaging and improve our understanding of seizure networks.

## CONFLICT OF INTEREST

The authors have no personal financial or institutional interest in the results described in this article.

## AUTHOR CONTRIBUTIONS

Justin Ke processed the data, defined ROIs and graphed the data; Lesley M. Foley acquired the MRI datasets; T. Kevin Hitchens established imaging protocols, supervised MR data acquisition, edited the manuscript; R. Mark Richardson provided the mTLE hippocampus samples and funding, edited the manuscript; Michel Modo conceived of the study, provided funding, oversaw the experiments, graphed the data, compiled figures and wrote the manuscript.

### Data Availability

The data that support the findings of this study are available from the corresponding author upon reasonable request.

## Supporting information


**Figure S1**: Mesoscale MR imaging of the human hippocampus. (a) The transverse view of a control hippocampus reveals a high mean diffusivity (MD/blue) in the granule cell layer (GCL) of the dentate gyrus (DG). In this plane, the classical hippocampal view with the pyramidal cell layer (PCL) surrounding the DG is evident in the head region. An overlay of MD and fractional anisotropy (FA/red) images helps to further define regions with high cellularity identified on MD and contrast this with regions with high FA value. Diffusion encoded color (DEC) FA images provide a further indication as to the primary direction of fiber tracts within different regions of interest (ROIs). Yellow crosshairs indicate the different planes presented here. (b) The saggital view further highlights the structural difference in organization of the head region versus the body and tail of the hippocampus. In this plane, a wavelike pattern is evident along the granule cell layer (GLC) in the body and tail region. (c) The coronal plane of the head reveals the classical view of hippocampal organization between different cell layers and the definition of different subfields of the pyramidal cell layer (PCL). In the coronoal plane, this organization is also evident along the hippocampal axis in the body and tail region. This is the preferred viewpoint to delineated different ROIs.

**Figure S2**: High resolution saggital and transverse views of a human hippocampus. (a) Three transverse images of hippocampal anatomy for comparison with tractography indicating fibers connecting different regions with each other. Long connections along the fimbra at the top of the sample can be seen and contrast with shorter connections along the pyramidal cell layer (PCL). (b) The laminar organization of the hippocampus is most evident in the saggital plane. It is also evident that not all connections are perpendicular to their cell layer, but in some case show crossing between different indendations in the granule cell layer (GCL), for instance.
