# Supplementary figures and images for: Ex vivo mesoscopic diffusion MRI correlates with seizure frequency in patients with uncontrolled mesial temporal lobe epilepsy
Source: Hum Brain Mapp. 2020 Jul 21;41(16):4529–48. doi: 10.1002/hbm.25139 (PMC7555080; doi:10.1002/hbm.25139)

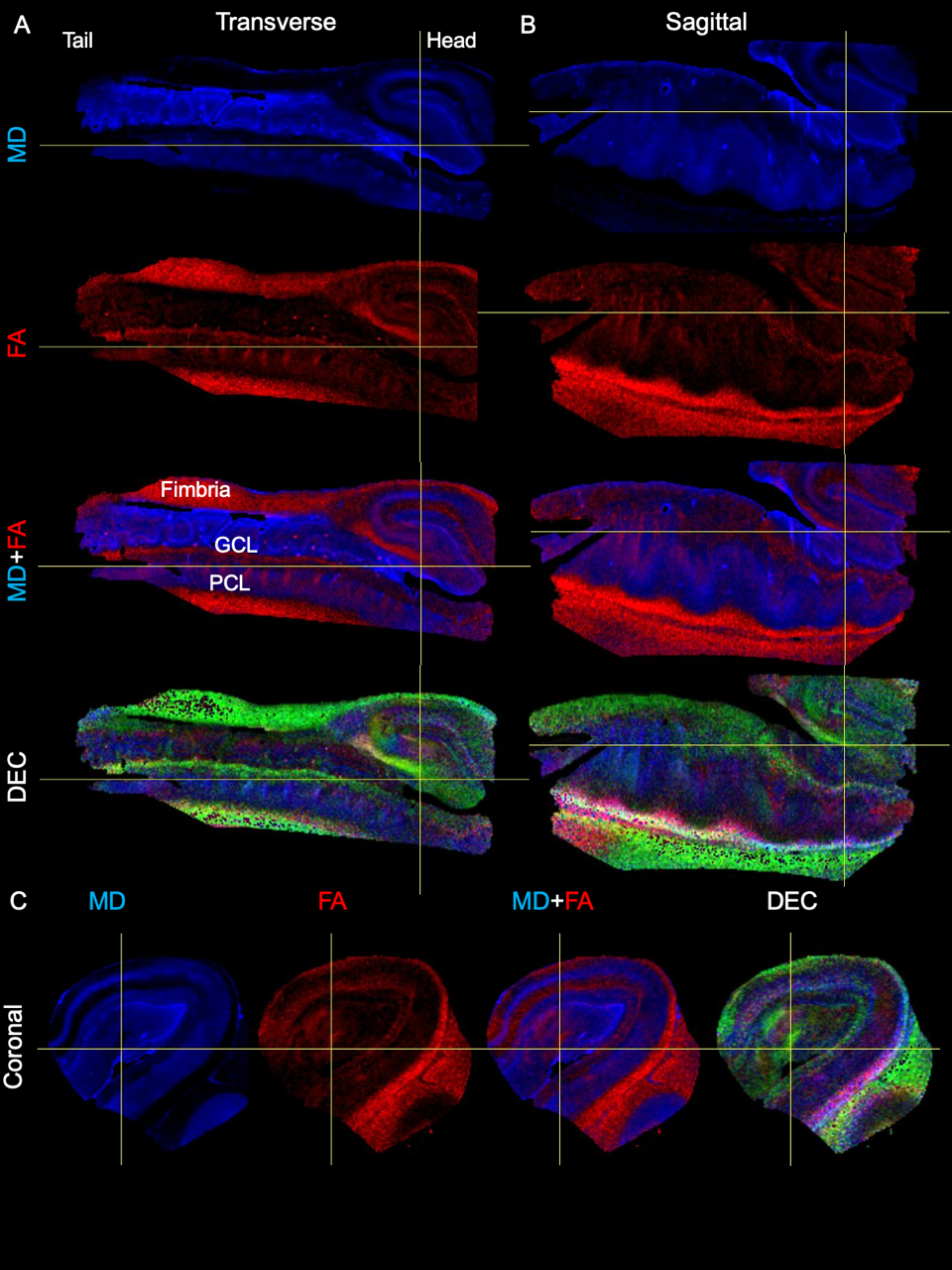

Supplement: Supplementary file 1 — Figure S1: Mesoscale MR imaging of the human hippocampus. (a) The transverse view of a control hippocampus reveals a high mean diffusivity (MD/blue) in the granule cell layer (GCL) of the dentate gyrus (DG). In this plane, the classical hippocampal view with the pyramidal cell layer (PCL) surrounding the DG is evident in the head region. An overlay of MD and fractional anisotropy (FA/red) images helps to further define regions with high cellularity identified on MD and contrast this with regions with high FA value. Diffusion encoded color (DEC) FA images provide a further indication as to the primary direction of fiber tracts within different regions of interest (ROIs). Yellow crosshairs indicate the different planes presented here. (b) The saggital view further highlights the structural difference in organization of the head region versus the body and tail of the hippocampus. In this plane, a wavelike pattern is evident along the granule cell layer (GLC) in the body and tail region. (c) The coronal plane of the head reveals the classical view of hippocampal organization between different cell layers and the definition of different subfields of the pyramidal cell layer (PCL). In the coronoal plane, this organization is also evident along the hippocampal axis in the body and tail region. This is the preferred viewpoint to delineated different ROIs. [file HBM-41-4529-s001.tif]

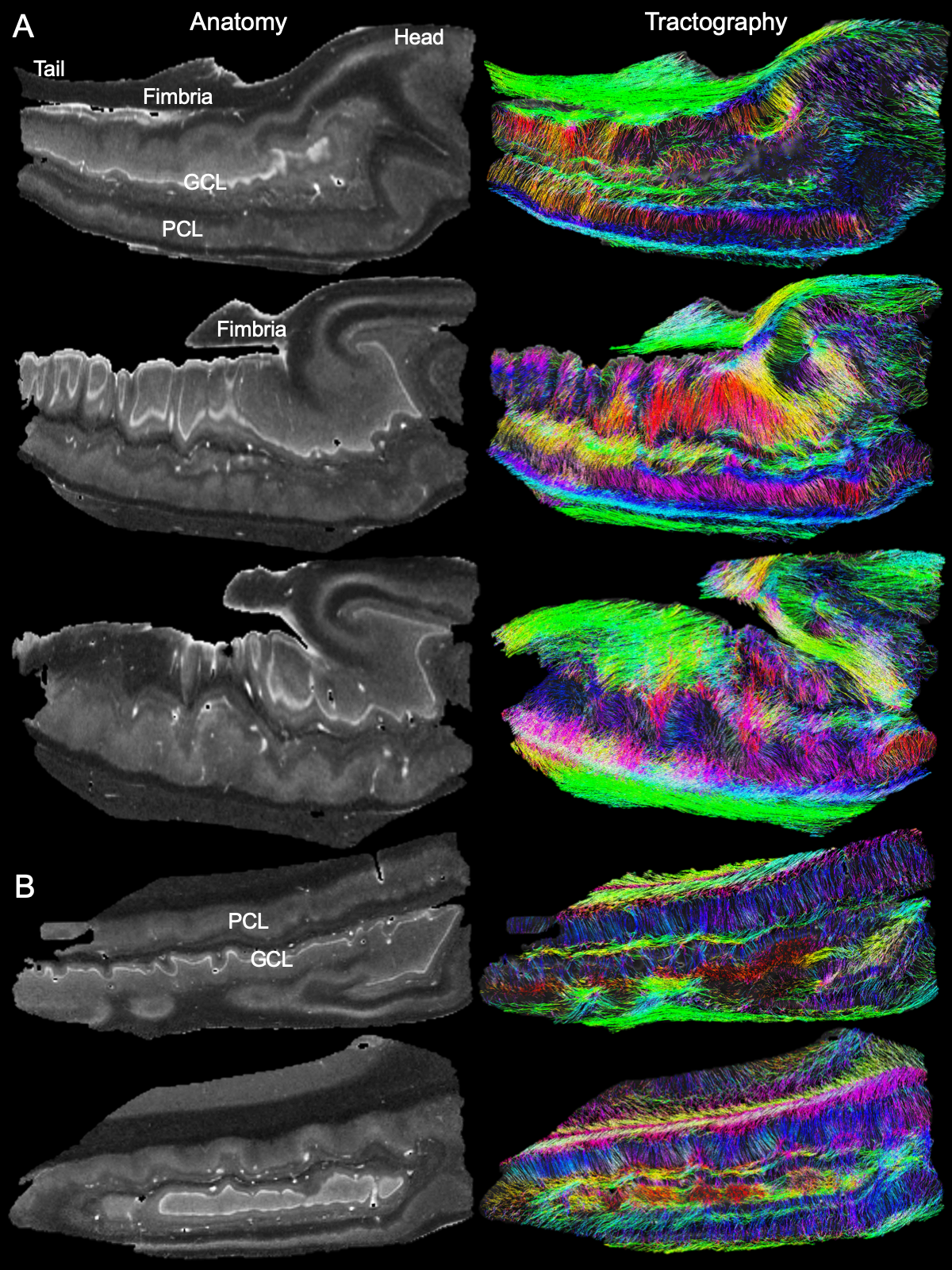

Supplement: Supplementary file 2 — Figure S2: High resolution saggital and transverse views of a human hippocampus. (a) Three transverse images of hippocampal anatomy for comparison with tractography indicating fibers connecting different regions with each other. Long connections along the fimbra at the top of the sample can be seen and contrast with shorter connections along the pyramidal cell layer (PCL). (b) The laminar organization of the hippocampus is most evident in the saggital plane. It is also evident that not all connections are perpendicular to their cell layer, but in some case show crossing between different indendations in the granule cell layer (GCL), for instance. [file HBM-41-4529-s002.tiff]
